# Disorders of Bulldogs under primary veterinary care in the UK in 2013

**DOI:** 10.1371/journal.pone.0217928

**Published:** 2019-06-12

**Authors:** Dan G. O’Neill, Alison M. Skipper, Jade Kadhim, David B. Church, Dave C. Brodbelt, Rowena M. A. Packer

**Affiliations:** 1 Pathobiology and Population Science, The Royal Veterinary College, Hawkshead Lane, North Mymms, Hatfield, Hertfordshire, United Kingdom; 2 Department of History, King’s College London, Strand, London, United Kingdom; 3 Clinical Sciences and Services, The Royal Veterinary College, Hawkshead Lane, North Mymms, Hatfield, Hertfordshire, United Kingdom; University of Lincoln, UNITED KINGDOM

## Abstract

The Bulldog is a popular companion breed in the UK despite widely reported disease predispositions. This study aimed to characterise the demography, mortality and common disorders of Bulldogs under veterinary care in the UK during 2013. VetCompass collates anonymised clinical data from UK primary-care veterinary practices for epidemiological research. The clinical records of all Bulldogs available in the VetCompass study dataset were reviewed manually in detail to extract the most definitive diagnoses recorded for all disorders that existed during 2013 and for all deaths. Bulldogs comprised 1621 (0.36%) of 445,557 study dogs. Bulldogs increased from 0.35% of the 2009 birth cohort to 0.60% in 2013. Median longevity was 7.2 years, which was lower in males (6.7 years) than females (7.9 years) (*P* = 0.021). The most prevalent fine-level precision disorders recorded were otitis externa (n = 206, prevalence 12.7%, 95% CI: 11.1–14.4), pyoderma (142, 8.8%, 95% CI: 7.4–10.2) and overweight/obesity (141, 8.7%, 95% CI: 7.4–10.2). The most prevalent disorder groups were cutaneous (n = 463, prevalence: 28.6%, 95% CI: 26.4–30.8), ophthalmological (292, 18.0%, 95% CI: 16.2–20.0), aural (211, 13.0%, 95% CI: 11.4–14.8), enteropathy (188, 11.6%, 95% CI: 10.1–13.3) and upper respiratory tract (171, 10.5%, 95% CI: 9.1–12.1). Provision of an evidence base on the most common disorders and causes of mortality within breeds can support owners, breeders and the veterinary profession to improve health and welfare within these breed.

## Introduction

Arguably the most iconic of British dog breeds, the Bulldog (British Bulldog) descends from dogs originally used for blood sports in the 16^th^ century [1]. These early bulldogs were small, thick-set dogs with powerful jaws that were used for bull-baiting [1]. After bull baiting was banned in 1835, the bulldog sank into obscurity, regarded as ‘a relic of a barbarous and bygone age’ [2, 3]. However, when the Kennel Club was founded in 1873, the rehabilitated Bulldog was among the first breeds it recognised [4]. The show Bulldog was selectively bred to resemble an idealised physical ‘breed standard’, in which various features, such as a head ‘the larger the better’ and a protruding lower jaw, were intended to define a dog suitably shaped for bull-baiting, despite the sport’s abolition [3]. Various subsequent changes, both in the breed standard and in its interpretation by breeders, have since further modified the shape of the Bulldog; the short ‘screw’ tail often seen today, for example, was a controversial novelty in the 1890s [5]. In recent years, the UK Kennel Club breed standard has been reworded to discourage extreme conformation [6].

In recent years, in line with a general rise in popularity of the brachycephalic (flat faced) breeds, the Bulldog has risen in popularity [7]. In 2017, the Bulldog was the sixth most common breed registered by the Kennel Club [8]. Although the Bulldog has not experienced such a dramatic surge in popularity as the French Bulldog and Pug have shown over the past decade, annual registration data from the UK Kennel Club still depicts over a two-fold increase in registrations during this time-scale, from 4543 (1.7% of all registrations) in 2008 to 9450 (3.9% of all registrations) in 2017 [9].

The distinctive physical appearance of brachycephalic breeds, including the Bulldog, is a key factor influencing their popularity [10]. However, some aspects of the Bulldog conformation, such as short muzzles and wrinkled faces, are associated with health problems [11–13]. Bulldogs are reported as predisposed to several health disorders, including brachycephalic obstructive airway syndrome (BOAS) [11, 14–16], dystocia [17], patellar luxation [18], seizures [19], corneal ulcers [12, 20] and spinal disease associated with vertebral malformations [21]. Indeed, Bulldogs have been reported with 39 breed predispositions to disease [22].

These disease predispositions are not new developments. There is ample archival evidence that a similar range of disorders already troubled Bulldogs over a century ago. Then, some Bulldog enthusiasts and judges, concerned that selection for exaggerated conformation had rendered the breed ‘a mere caricature’ of its bull-baiting ancestors [23] complained that the show Bulldog of c. 1900 had ‘a sadly shortened duration of life’ [24] and was beset by exercise intolerance and dystocia [23, 25]. They described features which today would be considered as linked to BOAS, such as nostrils ‘so small and pinched that it would be [a] hard job to pass a toothpick’ [26], upper jaws so short ‘as to render them incapable of getting a firm hold on anything more aggressive than a beefsteak’ [27], and that Bulldogs were ‘peculiarly susceptible’ to ‘heat apoplexy’ (hyperthermia) [28]. Canine veterinarians of that era recognised the increased risk of anaesthetising short-nosed breeds [29, 30]. Nonetheless, they advised early caesarian section for Bulldog bitches with dystocia, in the hope of at least saving the puppies, if not the bitch [29–32]. Breed activists, themselves often breeders, lamented these various issues. They urged judges not to award prizes to ‘cripples, deformities and grotesques … [with] exaggerated points’, and recommended breeding from ‘hardy and active’ dogs [33].

However, more than a century later, many Bulldogs are still struggling with similar difficulties [34, 35]. The UK Kennel Club lists the Bulldog as a Category 3 breed (the highest category) in its ‘Breed Watch’ system [36] with points of concern that focus on the airways, skin, tail, weight, eyes and gait [37].

Using veterinary clinical data from the VetCompass Programme [38], this study aimed to characterise the demography, longevity and common disorders of the general population of Bulldogs under veterinary care in the UK. The study additionally aimed to compare results between males and females, and specifically report the proportion of dogs recorded as blue or merle colour. Based on both the direct content of the current study and on comparability to other VetCompass breed studies, these results could support initiatives aimed at improving breeding and clinical management practices that ultimately contribute to better health and welfare of Bulldogs [39, 40].

## Materials and methods

The study population included all dogs under primary veterinary care at clinics participating in the VetCompass Programme during 2013. Dogs under veterinary care were defined as those with either a) at least one electronic patient record (EPR) (VeNom diagnosis term, free-text clinical note, treatment or bodyweight) recorded during 2013 or b) at least one EPR recorded both before and after 2013. VetCompass collates de-identified EPR data from primary-care veterinary practices in the UK for epidemiological research [38]. Data fields available to VetCompass researchers include a unique animal identifier along with species, breed, date of birth, colour, sex, neuter status and bodyweight, and also clinical information from free-form text clinical notes, summary diagnosis terms [41] and treatment with relevant dates.

A prevalence study design derived from the cohort clinical data of dogs registered at participating practices was used to estimate the one-year period prevalence of the most commonly diagnosed disorders [42]. Sample size calculations estimated that 1,696 dogs would be sampled to estimate a disorder that had 5% prevalence with 1% acceptable margin of error at a 95% confidence level (assuming Bulldogs comprised 0.3% of a UK population of 8 million dogs [43, 44]. Ethics approval was obtained from the RVC Ethics and Welfare Committee (reference number 2015/1369).

Dogs recorded as Bulldog breed at their final available record were categorised as Bulldog and all remaining dogs were categorised as non-Bulldog. Breed classification was thus ultimately based on owner and/or veterinary practice identification that could be refined over time as the dog revisited the practice. No distinction was made between show, Kennel Club registered or unregistered Bulldogs. Animals that were recorded as entirely or partly blue were categorised as ‘blue’. *Adult Bodyweight* described the mean bodyweight (Kg) recorded from all bodyweight data for dogs aged over 18 months at the time of weighing and was categorised into 6 groups (< 20.0, 20.0 to < 24.0, 24.0 to < 28.0, 28.0 to < 32.0, 32.0 to < 36.0, ≥ 36.0). N*euter* described the status of the dog (entire or neutered) at the final EPR. *Age* described the age (years) at the final date under veterinary care during 2013 (December 31^st^, 2013) and was categorised into 8 groups (< 1.0, 1.0 to < 2.0, 2.0 to < 4.0, 4.0 to < 6.0, 6.0 to < 8.0, 8.0 to < 10.0, 10.0 to < 12.0, ≥ 12.0).

The list of unique Bulldog animal identification numbers was randomly ordered and the clinical records of all animals were reviewed manually in detail to extract the most definitive diagnoses recorded for all disorders that existed during 2013 [15]. Elective (e.g. neutering) or prophylactic (e.g. vaccination) clinical events were not included. No distinction was made between pre-existing and incident disorder presentations. Disorders described within the clinical notes using presenting sign terms (e.g. ‘vomiting’ or 'vomiting and diarrhoea'), but without a formally recorded clinical diagnostic term, were included using the first sign listed (e.g. vomiting). These disorders included in the study did not need to be the reason that the dog presented for veterinary care; awareness of the existence of many of these disorders was first raised as part of veterinary examinations during routine visits for prophylactic care such as vaccination or during visits that were precipitated by an altogether different condition. Mortality data (recorded cause, date and method of death) were extracted on all deaths at any date during the available EPR data. The extracted diagnosis terms were mapped to a dual hierarchy of diagnostic precision for analysis: fine-level precision and grouped-level precision as previously described [15]. Briefly, fine-level precision terms described the original extracted terms at the maximal diagnostic precision recorded within the clinical notes (e.g. *inflammatory bowel disease* would remain as *inflammatory bowel disease*). Grouped-level precision terms mapped the original diagnosis terms to a general level of diagnostic precision (e.g. *inflammatory bowel disease* would map to *gastro-intestinal*). This approach of reporting disorders at two levels of precisions aimed to accommodate for the varying depths of clinical precision across the breadth of primary care clinical caseloads.

Following data checking for internal validity and cleaning in Excel (Microsoft Office Excel 2013, Microsoft Corp.), analyses were conducted using Stata Version 13 (Stata Corporation). The sex, neuter status, age, colour and adult bodyweight for Bulldogs under veterinary care during 2013 were described. Annual proportional birth rates described the relative proportion of Bulldogs compared with all dogs that were born in each year from 2008–2013 from the cohort that were under veterinary care in 2013. All-age bodyweight data with their associated dates were used to generate individual bodyweight growth curves for male and female Bulldogs by plotting age-specific bodyweights and were overlaid with a cross medians line plot using the Stata *mband* command.

One-year (2013) period prevalence values were reported along with 95% confidence intervals (CI) that described the probability of diagnosis at least once during 2013. The CI estimates were derived from standard errors based on approximation to the normal distribution for disorders with ten or more events [45]. The median age at the end of the study date range was reported for affected animals. Prevalence values were reported overall and also separately for males and females. The chi-square test was used to compare categorical variables and the Students t-test or Mann-Whitney U test to compare continuous variables as appropriate [45]. Statistical significance was set at the 5% level.

From a qualitative research perspective [46], this paper discussed the health concerns that were written about for the Bulldog during its previous peak of popularity (around 1900) in relation to the main concerns of the modern Bulldog. This archival research was carried out through a survey of publications (newspapers, journals and books) serving the British dog breeding and veterinary communities at this time. If was not possible to compare the health status between these periods directly because there was no comparable quantitative data resource available for the breed in 1900.

## Results

### Demography and mortality

**Fig 1:**
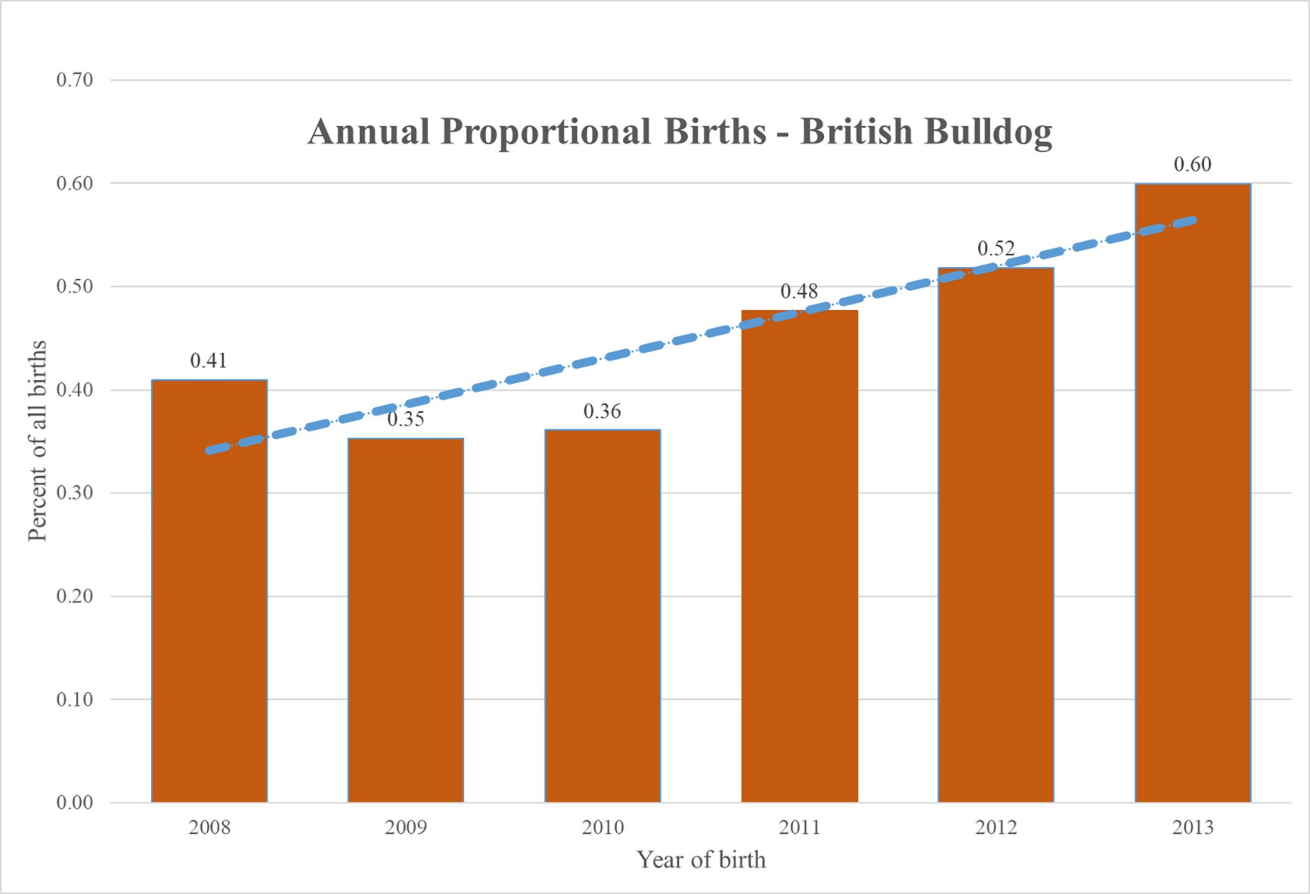
Annual proportional birth rates (2008–2013) for Bulldogs in the UK. The analysis included 1,621 Bulldogs from 455,557 dogs attending UK primary-care veterinary clinics participating in the VetCompass Programme.

The study population of 455,557 dogs from 304 clinics in the VetCompass database under veterinary care during 2013 included 1,621 (0.36%) Bulldogs. This is a lower percentage than indicated by Kennel Club registration figures, but practice data additionally includes unregistered dogs of all breeds, including those, such as ‘designer’ and other crossbreeds, which are ineligible for Kennel Club registration [15]. Annual proportional birth rates showed gradually rising popularity of Bulldogs in the UK, ranging from 0.35% of all puppy births in 2009 to 0.60% in 2013 (Fig 1).

**Fig 2:**
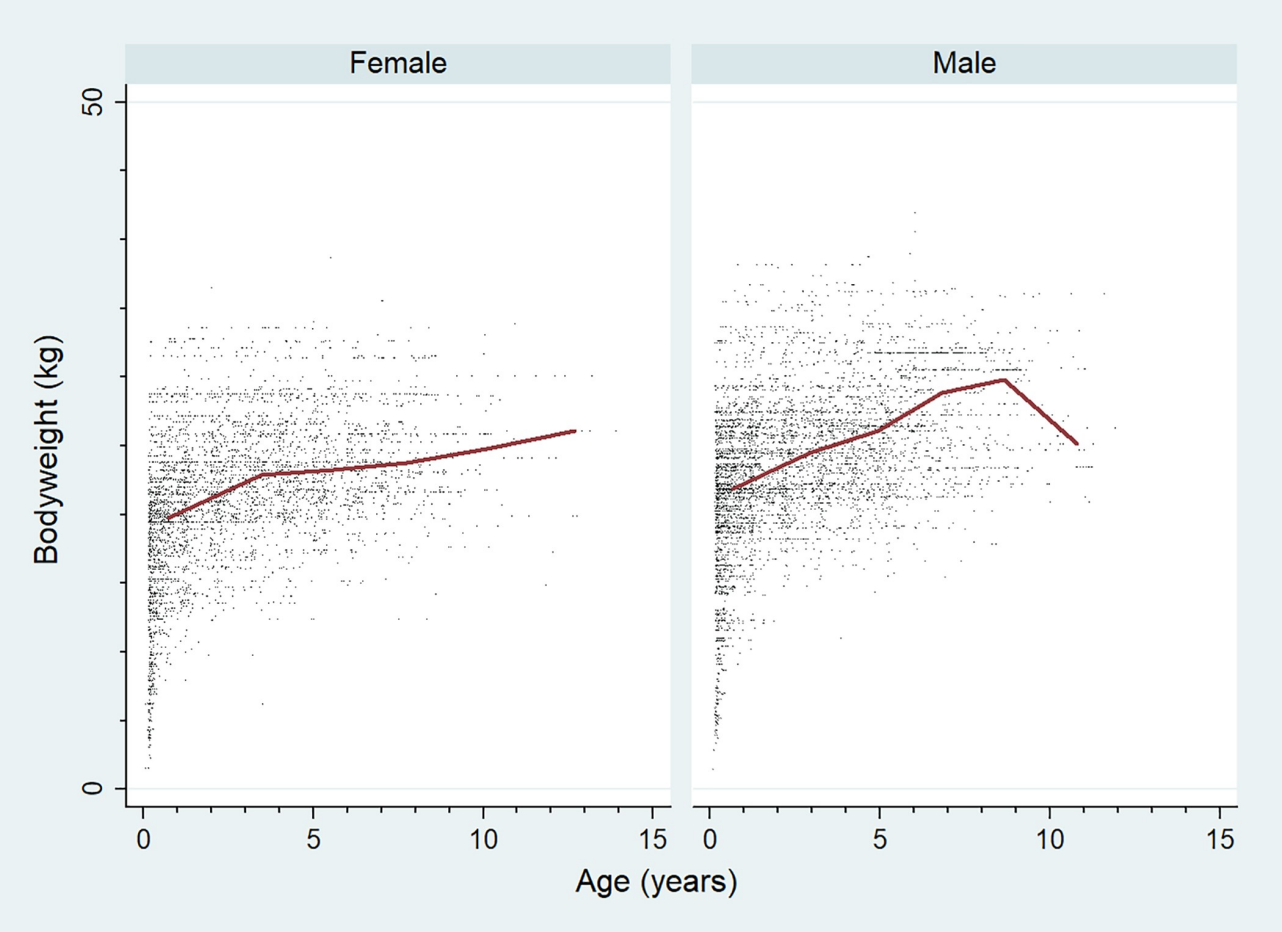
Bodyweight growth curves for female and male Bulldogs in the UK. The analysis included 3,974 bodyweight values from 662 female Bulldogs and 5,163 bodyweight values from 690 male Bulldogs attending UK primary-care veterinary clinics participating in the VetCompass Programme. The bodyweight growth curves are overlaid with a cross medians line plot.

Of the Bulldogs with information available, 805 (49.9%) were female and 410 (25.8%) were neutered. Neuter status did not differ between females and males (25.3% versus 26.6%, *P* = 0.550). Eight dogs (0.5%) were recorded as blue (partly or completely) and there were no dogs recorded as merle. The median age of the Bulldogs overall was 2.3 years (IQR 1.1–4.5, range 0.1–14.0). The overall mean adult bodyweight was 26.0 kg (standard deviation [SD] 4.7 kg). The mean adult bodyweight of males (27.6 kg, SD 4.6 kg) was heavier than females (24.3 kg, SD 4.1 kg) (*P* < 0.001) (Table 1). The median bodyweight across all ages for males (23.9 kg, IQR: 21.1–27.3, range: 1.5–42.0) was higher than for females (21.7 kg, IQR: 18.6–24.7, range: 1.6–38.7) (P < 0.001). Bodyweight growth curves based on 3,974 bodyweight values from 662 females and 5,163 bodyweight values from 690 males across all ages showed that Bulldog puppies grow rapidly during their first year but progressive with slower bodyweight increases for most of their adult lives (Fig 2). Data completeness varied across the variables assessed: sex 99.5%, neuter 97.9%, age 97.7%, colour 92.8% and all-age bodyweight 83.8%.

**Table 1:** Demography of Bulldogs under primary veterinary care at practices participating in the VetCompass Programme in the UK from January 1^st^, 2013 to December 31^st^, 2013 (n = 1,621).

Variable	Category	Count*	Percent
Sex	Female	805	49.9
	Male	808	50.1
Female neuter	Entire	596	74.7
	Neutered	202	25.3
Male neuter	Entire	573	73.4
	Neutered	208	26.6
Colour	Blue (partly or completely)	8	0.5
	Merle	0	0.0
	Not blue or merle	1,497	99.5
Female adult bodyweight (aged ≥ 18 months) (kg)	< 20.0	68	14.2
	20.0 to < 24.0	169	35.2
	24.0 to < 28.0	161	33.5
	28.0 to < 32.0	59	12.3
	32.0 to < 36.0	20	4.2
	≥ 36.0	3	0.6
Male adult bodyweight (aged ≥ 18 months) (kg)	< 20.0	14	2.7
	20.0 to < 24.0	98	18.9
	24.0 to < 28.0	183	35.3
	28.0 to < 32.0	131	25.3
	32.0 to < 36.0	67	12.9
	≥ 36.0	25	4.8
Age (years)	< 1.0	372	23.5
	1.0 to < 2.0	333	21.0
	2.0 to < 4.0	398	25.1
	4.0 to < 6.0	271	17.1
	6.0 to < 8.0	125	7.9
	8.0 to < 10.0	62	3.9
	10.0 to < 12.0	19	1.2
	≥ 12.0	3	0.2

* Results from dogs with available data.

There were 181 deaths recorded during the study. The median longevity of Bulldogs overall was 7.2 years (IQR 4.9–9.3, range 0.0–14.1). The median longevity of females (7.9 years, IQR 4.9–10.2, range 0.7–14.1, n = 73) was greater than males (6.7 years, IQR 4.4–9.1, range 0.0–11.9, n = 107) (*P* = 0.021). The median longevity of neutered animals (8.0 years, IQR 5.8–10.3, range 1.2–14.1, n = 66) was greater than entire animals (6.7 years, IQR 4.3–9.0, range 0.0–13.0, n = 113) (*P* = 0.003). The cause of death was not recorded for 71 (39.2%) of deaths. Of the 110/181 dogs with a recorded cause of death, the most common causes of death described at a grouped-precision level were heart disease (n = 13, prevalence 11.8%), neoplasia (12, 10.9%) and brain disorder (10, 9.1%) (Table 2).

**Table 2:** Grouped causes of mortality in Bulldogs with a recorded cause of death under primary-care veterinary at UK practices participating in the VetCompass Programme from January 1^st^, 2013 to December 31^st^, 2013 (n = 110).

Grouped disorder term	Count	Percent	95%CI*
Heart disease	13	11.8	6.4–19.4
Neoplasia	12	10.9	5.8–18.3
Brain disorder	10	9.1	4.4–16.1
Collapsed	7	6.4	2.6–12.7
Lower respiratory tract disorder	7	6.4	2.6–12.7
Mass-associated disease	7	6.4	2.6–12.7
Undesirable behaviour	6	5.5	2.0–11.5
Upper respiratory tract disease	6	5.5	2.0–11.5
Other	42	38.2	29.1–47.9
Total	110		

* CI confidence interval

### Disorder prevalence

The EPRs of all 1,621 Bulldogs were manually examined to extract all recorded disorder data for 2013. There were 1,148 (70.8%) Bulldogs with at least one disorder recorded during 2013 while the remaining 29.2% had no disorder recorded and either presented for prophylactic management only or did not present at all during 2013. The median annual disorder count per Bulldog during 2013 was 1 disorder (IQR 0–3, range 0–15) and was higher in males (median 1.5, IQR 0–3, range 0–15) compared with females (median 1, IQR 0–3, range 0–14) (*P* = 0.006).

The study included 3,298 unique disorder events recorded during 2013 that encompassed 296 distinct fine-level disorder terms. The most prevalent fine-level precision disorders recorded were otitis externa (n = 206, prevalence 12.7%, 95% CI: 11.1–14.4), pyoderma (142, 8.8%, 95% CI: 7.4–10.2), overweight/obesity/ (141, 8.7%, 95% CI: 7.4–10.2), skin fold dermatitis (126, 7.8%, 95% CI% 6.5–9.2), overlong nails (119, 7.3%, 95% CI 6.1–8.7) and prolapsed gland of third eyelid (cherry eye) (110, 6.8%, 95% CI% 5.6–8.1). Males had higher prevalence than females for 4 of the 29 most common fine-level precision disorders (pyoderma, interdigital cyst, atopic dermatitis and aggression) while females had higher prevalence for 2 disorders (periodontal disease and overweight/obesity). There were 50 dogs (3.1% prevalence, 95% CI: 2.3–4.0) recorded with a condition that was paradoxically labelled as ‘normal for the breed’ (Table 3).

**Table 3:** Prevalence of the most common disorders at a *fine-level of diagnostic precision* recorded in Bulldogs (n = 1,621) attending UK primary-care veterinary practices participating in the VetCompass Programme from January 1^st^, 2013 to December 31^st^, 2013. The P-value reflects prevalence comparison between females and males. The median age described the age of diagnosed cases on the end of the study date range.

Fine-level disorder	Count	Overall prevalence %	95% CI*	Female prevalence %	Male prevalence %	P-Value	Median age at diagnosis (years)
Otitis externa	206	12.7	11.1–14.4	11.6	14.0	0.143	3.8
Pyoderma	142	8.8	7.4–10.2	6.5	11.1	**0.001**	2.4
Overweight/ obesity	141	8.7	7.4–10.2	10.7	6.8	**0.006**	2.7
Skin fold dermatitis	126	7.8	6.5–9.2	7.1	8.5	0.275	2.4
Overlong nails	119	7.3	6.1–8.7	7.0	7.8	0.518	2.8
Prolapsed gland of third eyelid	110	6.8	5.6–8.1	6.2	7.4	0.333	1.2
Cryptorchidism (males only)	45	5.6	4.1–7.4	~	~	~	1.1
Conjunctivitis	88	5.4	4.4–6.6	4.8	6.1	0.281	
Pododermatitis	88	5.4	4.4–6.6	4.5	6.4	0.083	4.0
Alopecia	86	5.3	4.3–6.5	4.5	6.2	0.125	2.2
Diarrhoea	79	4.9	3.9–6.0	4.1	5.7	0.138	1.1
Pyotraumatic dermatitis	70	4.3	3.4–5.4	3.5	5.2	0.090	2.2
Interdigital cyst	60	3.7	2.8–4.7	2.2	5.2	**0.002**	3.7
Entropion	58	3.6	2.7–4.6	3.9	3.3	0.583	2.8
Vomiting	58	3.6	2.7–4.6	2.7	4.5	0.063	1.5
Brachycephalic obstructive airway syndrome (BOAS)	57	3.5	2.7–4.5	3.1	4.0	0.353	2.7
Atopic dermatitis	57	3.5	2.7–4.5	2.1	5.0	**0.002**	3.6
Corneal ulceration	51	3.1	2.4–4.1	3.0	3.3	0.679	2.8
Normal for breed	50	3.1	2.3–4.0	2.4	3.8	0.087	1.3
Prognathism	47	2.9	2.1–3.8	3.6	2.2	0.101	1.0
Umbilical hernia	45	2.8	2.0–3.7	2.9	2.6	0.750	1.1
Lameness	44	2.7	2.0–3.6	2.1	3.3	0.130	1.8
Malassezia dermatitis	40	2.5	1.8–3.3	2.0	3.0	0.204	4.2
Aggression	39	2.4	1.7–3.3	1.6	3.2	**0.036**	4.1
Keratoconjunctivitis sicca	36	2.2	1.6–3.1	2.1	2.4	0.745	6.1
Periodontal disease	34	2.1	1.5–2.9	2.9	1.4	**0.037**	5.5
Demodicosis	33	2.0	1.4–2.8	1.4	2.7	0.054	1.3
Distichiasis	32	2.0	1.4–2.8	2.1	1.9	0.713	2.6
Upper respiratory tract infection	25	1.5	1.0–2.2	1.6	1.5	0.833	1.0

* CI confidence interval

The study included 52 distinct grouped-level precision disorder terms. The most prevalent grouped-level precision disorders were cutaneous (n = 463, 28.6%, 95% CI: 26.4–30.8), ophthalmological (292, 18.0%, 95% CI: 16.2–20.0), aural (211, 13.0%, 95% CI: 11.4–14.8) and enteropathy (188, 11.6%, 95% CI: 10.1–13.3). Males had higher prevalence than females for cutaneous and enteropathy disorders while females had higher prevalence for overweight/obesity (Table 4).

**Table 4:** Prevalence of the most common *grouped-level disorders* recorded in Bulldogs (n = 1,621) attending UK primary-care veterinary practices participating in the VetCompassProgramme from January 1^st^, 2013 to December 31^st^, 2013. The P-value reflects prevalence comparison between females and males. The median age described the age of cases on the end of the study date range.

Grouped-level disorder	Count	Overall prevalence	95% CI*	Female prevalence %	Male prevalence %	P-Value	Median age (years)
Cutaneous	463	28.6	26.4–30.8	25.5	31.9	**0.004**	2.5
Ophthalmological	292	18.0	16.2–20.0	17.6	18.6	0.630	1.8
Aural	211	13.0	11.4–14.8	11.8	14.4	0.128	4.0
Enteropathy	188	11.6	10.1–13.3	9.8	13.5	**0.021**	1.3
Upper respiratory tract	171	10.5	9.1–12.1	9.6	11.6	0.177	1.4
Overweight/obesity	141	8.7	7.4–10.2	10.7	6.8	**0.006**	2.7
Claw/nail	136	8.4	7.1–9.8	7.7	9.2	0.292	2.7
Musculoskeletal	110	6.8	5.6–8.1	6.3	7.3	0.441	3.6
Male reproductive (males only)	55	6.8	5.2–8.8	~	~	~	1.2
Female reproductive (females only)	45	5.6	4.1–7.4	~	~	~	2.9
Parasitic	74	4.6	3.6–5.7	4.2	5.0	0.485	1.1
Behavioural	65	4.0	3.1–5.1	3.4	4.7	0.168	3.0
Neoplastic	58	3.6	2.7–4.6	3.1	4.1	0.291	4.0
Urinary	58	3.6	2.7–4.6	4.0	3.2	0.414	2.4

* CI confidence interval

## Discussion

This study of over one thousand six hundred animals is the largest analysis to date in the UK of breed health in Bulldogs based on primary-care veterinary records. The results highlight a gradual rise in Bulldog ownership in the UK. Bulldogs comprised over 0.6% of all dogs born in 2013 attending the participating veterinary practices, although some of these dogs may have been born outside the UK. Given that the longevity data presented in this paper shows a relative short median longevity for Bulldogs of 7.2 years, it is possible that their rising ownership may be exaggerated as an artefact of their short lifespan that promotes left truncation of the data [47, 48]. However, annual registration data from the UK Kennel Club similarly depicts a two-fold increase in registrations over the past ten years, so the popularity of Bulldogs, along with other small-medium sized, flat-faced breeds, appears to be truly rising. This may be because potential owners consider them physically appealing but this increasing popularity is concerning because several of the most common disorders in Bulldogs are linked with their physical conformation [10].

Much of the previous evidence on health issues in Bulldogs was derived from referral, insurance and especially owner questionnaire data sources [49]. In contrast, the current study relies on de-identified clinical data recorded by primary-care veterinary teams and therefore offers a new perspective on the health issues that can supplement these other sources to build a more complete picture of breed health [50]. Research using veterinary clinical records that are recorded contemporaneously at the time of the clinical event and with no prior awareness of the subsequent specific research topics reduces recall and selection biases [49]. Results from breed-health studies such as the current study are contributing to modern breed health strategies such as those of the Kennel Club’s Breed Health and Conservation Plans project [40] and the strategic framework aims of the Brachycephalic Working Group [39].

The Kennel Club only permits registration of Bulldogs of a defined list of fifteen colourings: include brindle, fawn, red, red fawn, red brindle, as well as each of these colours combined with white [51]. The Kennel Club does not recognise blue, an autosomal recessive dilution which produces a slate-grey colour phenotype or merle, the blotched grey and black colouration seen in dogs heterozygous for the merle gene, colours in Bulldogs [52]. Merle is associated with both auditory and ophthalmologic abnormalities, particularly in homozygotes [52, 53]. Although there have been reports of a rise in public demand for blue (and, very recently, merle) Bulldogs [54], the current study reported that 0.5% were blue and 0.0% were merle in the general Bulldog population in 2013.

The 7.2 years median longevity of the Bulldog reported here was almost five years shorter than the 12.0 years median reported previously for dogs overall in England [55]. It is interesting to note that 7 years was still described as ‘quite an old age for show Bulldogs’ in 1901 [56]. Common causes of death in the current study included heart disease, neoplasia, brain disorders and respiratory tract (lower and upper) disorders. The relatively short median longevity of this breed may be a reflection of a generally high disorder burden, with the majority of Bulldogs (70.8%) recorded to have at least one disorder during 2013. These veterinary-derived results are markedly higher than the disorder prevalence estimated in an owner self-reported study in 2017, where only half (48.11%) of Bulldogs were reported as having 1 or more disorders [16]. This disparity may in part be due to the different data collection methodologies, with owners being susceptible to recognition biases when they consider disorders such as dyspnoea as ‘normal for the breed’ and therefore fail to acknowledge these as true health problems in their dog [57]. However, such normalising phenomena are also shown by veterinary surgeons; in the present study, 3.1% of dogs had a condition that was classified as ‘normal for the breed’ recorded in their clinical notes. Such normalisation may paradoxically result in underestimated prevalence figures for disorders that are common in a breed. While desensitisation of veterinary professionals to breed-related problems is problematic from a research perspective, it may also lead to potential under-treatment of clinical patients.

The most common disorders identified in Bulldogs in the current study were otitis externa, pyoderma, overweight/obesity and skin fold dermatitis. These results can provide a framework to identify health priorities in Bulldogs that can contribute positively to improved health and welfare within the breed. Some health distinctions were also identified between the sexes in this population of Bulldogs. Female Bulldogs lived over one year longer than males, with a similar female longevity advantage previously recorded in breeds such as the Rottweiler [58] and German Shepherd Dog [59] but not in others such as the French Bulldog [60]). It is unclear why a consistent sex-based longevity effect does not exist across breeds but differences may be explained by differing sex-associated disorder prevalence, inbreeding effects and early deaths across breeds [61–63]. Marked sex differences in disorder prevalence were observed in the current study, with males more likely than females to be diagnosed with 5 of the 29 most common fine-level precision disorders, and 2 of the 14 most common grouped-level precision disorders. The only disorders at fine- or grouped-level precision that were more common in female dogs were overweight/obesity. Whether this apparent female predisposition to overweight/obesity is a biological effect (e.g. hormonal), husbandry-related (e.g. owner influence) or assessment based (e.g. differing use of body condition scoring between males and females) is not clear [64]. These additional sex-based prevalence data can highlight those disorders that would benefit from special focus within specific sexes in order to contribute to improved Bulldog health and welfare as well as assisting decision-making by veterinarians and owners on the most appropriate sex selection when first deciding on puppy purchase [15, 65].

Skin disorders were the most common grouped-level category in the Bulldog, with an overall prevalence of 28.6%. At the fine-level diagnosis level, common skin disorders included pyoderma (8.8%), skin fold dermatitis (7.8%), pododermatitis (5.4%), alopecia (5.3%), pyotraumatic dermatitis (4.3%) and atopic dermatitis (3.5%) among the top 20 most common fine-level disorders in the breed. Skin disease is well recognised as a problem in Bulldogs; as long ago as 1897, concern for the high ‘general prevalence of eczema’ in the breed occasioned specific comment [66]. Kennel Club Breed Watch raises points of concern related to the skin, including hair loss or scarring from previous dermatitis, and lists several anatomical features of Bulldogs that may predispose to skin fold dermatitis including heavy overnose wrinkle (roll), inverted tail and tight tail [37]. Skin fold dermatitis may occur at any body site where excessive skin wrinkling causes skin-on-skin contact, including the facial region of brachycephalic dogs or in skin folds around absent, short or screw-tails [67]. High levels of fold-related skin disease have been documented in other small brachycephalic breeds, such as the Pug and French Bulldog [60, 68], which share the characteristic facial wrinkling and short or screw-tails features of the Bulldog. The Kennel Club breed standard for the Bulldog currently describes facial folds as “*Over nose wrinkle*, *if present*, *whole or broken*, *must never adversely affect or obscure eyes or nose*” [51], while the American Kennel Club standard more explicitly encourages a wrinkled look; “*The head and face should be covered with heavy wrinkles*” [69]. With skin conditions so prevalent in the Bulldog, it would be sensible to avoid breeding for exaggerated skin fold conformations that promote high welfare risks without advantages for the dog.

In addition to skin disorders that are directly related to conformation, atopic dermatitis (3.5%) featured in the top 20 most common disorders of the Bulldog in agreement with previous reports in the veterinary dermatology literature that the Bulldog breed is predisposed to canine atopic dermatitis [70]. Additionally, the most common fine-level disorder was otitis externa (12.7%). With previous research demonstrating that 43% of otitis externa cases result from underlying atopic dermatitis [71], and 59% of recurrent pyoderma cases reported as sequelae to allergic skin disease [72] it is possible that some or many of these otitis and pyoderma cases are associated with allergic skin disease pathology.

Ophthalmological disorders were the second most common grouped-level disorder of Bulldogs (18.0%), with prolapsed nictitans gland (6.8%), conjunctivitis (5.4%), entropion (3.6%) and corneal ulceration keratitis (3.1%) in the top 20 most common fine-level disorders in the breed. Many of these problems also have a long history of being reported in the breed; entropion, ectropion and conjunctivitis were described as prevalent in Bulldogs in 1908 [73]. A previous VetCompass study of corneal ulcers identified Bulldogs as the fifth most commonly affected breed (prevalence: 2.41%) and with six-times higher odds of corneal ulceration compared with crossbreeds [20]. In a prospective clinical study, 18% of Bulldogs were affected by corneal ulcers [12], with conformational risk factors for corneal ulceration identified including the presence of a nasal fold and brachycephalic skull shape which are both characteristic features of the Bulldogs [12]. Although Bulldogs do not share the same exophthalmic eye conformation as other brachycephalic breeds predisposed to corneal ulcers such as the Pug [12, 20], the marked nasal skin fold of the Bulldog may lead to medial canthal entropion and nasal fold trichiasis due to direct contact between the nasal fold and cornea. The Kennel Club (UK) has recently taken efforts to reduce this problem, with Breed Watch points of concern for the Bulldog including ‘*sore eyes due to damage or poor eyelid conformation*’, ‘*Excessive amounts of loose facial skin with conformational defects of the upper and/or lower eyelids so that the eyelid margins are not in normal contact with the eye*’ and ‘*Heavy overnose wrinkle (roll)*’ [37].

Upper respiratory tract (URT) disorders were commonly recorded in Bulldogs, being the fifth most common disorder at grouped-level diagnostic precision and affecting 10.5% of dogs. At fine-level diagnostic precision, the most common respiratory tract disorder was brachycephalic obstructive airway syndrome (BOAS) (3.5%; median age 2.7 years). With clinical signs that include chronic breathlessness, exercise intolerance, eating difficulties and disrupted sleeping including periods of apnoea, BOAS is a disorder of major animal welfare concern that adversely affects animals both while awake and asleep [74]. Archival sources describe a similar presentation extending back over a century. In 1902, a breed activist complained that the new-style Bulldog was ‘so exaggerated that … [it] can only move with difficulty, and pants painfully with the least exertion’ [24]. The apparently low prevalence of BOAS recorded in the current retrospective study contrasts with much higher prevalence reported in other prospective and disorder-specific studies. Sixty-three percent of Bulldogs attending a UK veterinary referral hospital were diagnosed with BOAS based on clinical history, owner questionnaire and clinical examination [11]. A study using whole-body barometric plethysmography reported that 84.8% of tested Bulldogs were affected by BOAS to some extent, with 44.0% exhibiting clinically relevant disease [75]. Finally, an earlier VetCompass study reported that 19.5% of Bulldogs had at least one URT disorder over a 4.5 year study period [76], compared to the one year period of the current study. Recognition of BOAS by both owners and veterinarians may also be hampered by perceptions that this disorder is ‘normal for the breed’; indeed, nearly 60% of owners of dogs affected by BOAS did not perceive that their dog had a breathing problem [57, 77]. Efforts to mitigate airway disease in the breed have been introduced via the Kennel Club Breed Watch scheme, where points of concern cover dogs showing respiratory distress including difficulty in breathing or laboured breathing, and pinched nostrils (a risk factor for BOAS [75]) [37]. Such measures, however, only directly influence those breeders who engage with Kennel Club initiatives and it may take some time for such progress to spill over to the wider population of dogs that are not registered with the Kennel Club. Moreover, the data used for the current study were collected relatively soon after the 2009 launch of Breed Watch, and therefore describes a Bulldog population that may yet not have benefitted much by its recommendations. As BOAS is intimately linked with conformation, major reductions in prevalence are unlikely without accompanying conformational change and therefore high levels of awareness and willingness to engage is critically needed from breed communities to ensure success [11].

This study had some limitations. Because primary-care practice data seldom records such information, the study did not directly assess the conformation of the dogs involved nor differentiate between the show, registered and unregistered populations. Therefore, while comparison of disease levels between these groups would be illuminating, it was consequently beyond the scope of this paper. Research using primary-care veterinary clinical data has some inherent limitations that have been previously reported [78]. Studies based on reviews of animal medical records may under-estimate true disease burdens by predominantly including those more severely affected animals that warrant veterinary management and there may be reduced reporting of less severely affected animals that may be less likely to be clinically presented [79]. Clinical variants of some diseases may be recorded using several distinct disorder terms and therefore the combined prevalence for these diseases may be fragmented into separate prevalence values for each of multiple more-specific diagnostic term, giving the illusion of lower prevalence [15]. The practices participating in the study were a convenience sample and may not be fully representative of the overall veterinary practice structure and caseloads in in the UK.

## Conclusions

This study of over sixteen hundred Bulldogs documents rising ownership of this breed in the UK and provides important disorder prioritisation based on the general population of Bulldogs. The most common disorders in Bulldogs were otitis externa, pyoderma and overweight/obesity. The skin was the most affected body region, with pyoderma, skin fold dermatitis and pododermatitis among the top ten most common disorders of Bulldogs. Many common disorders of Bulldogs are linked with their appearance, including skin fold disease, BOAS and corneal ulceration. By contextualising these data within a longer historical timeframe, this paper observes that the high frequency of conformation-related disease in Bulldogs is a long-standing problem that also led breeders themselves to question conformational breeding goals over a century ago. These results provide a framework of health priorities for Bulldogs and therefore can contribute positively to improved health and welfare within the breed.

## Abbreviations

BOAS: brachycephalic obstructive airway syndrome
CI: confidence interval
EPR: electronic patient record
IQR: interquartile range
OR: odds ratio
URT: upper respiratory tract
